# Gene polymorphisms against DNA damage induced by hydrogen peroxide in leukocytes of healthy humans through comet assay: a quasi-experimental study

**DOI:** 10.1186/1476-069X-9-21

**Published:** 2010-05-05

**Authors:** Ana L Miranda-Vilela, Penha CZ Alves, Arthur K Akimoto, Graciana S Lordelo, Carlos A Gonçalves, Cesar K Grisolia, Maria N Klautau-Guimarães

**Affiliations:** 1 Departamento de Genética e Morfologia, Laboratório de Genética, Instituto de Ciências Biológicas, Universidade de Brasília, Brasília/DF, Brasil; 2 Departamento de Ciências Fisiológicas, Laboratório Integrado, Instituto de Ciências Biológicas, Universidade de Brasília, Brasília/DF, Brasil

## Abstract

**Background:**

Normal cellular metabolism is well established as the source of endogenous reactive oxygen species which account for the background levels of oxidative DNA damage detected in normal tissue. Hydrogen peroxide imposes an oxidative stress condition on cells that can result in DNA damage, leading to mutagenesis and cell death. Several potentially significant genetic variants related to oxidative stress have already been identified, and angiotensin I-converting enzyme (ACE) inhibitors have been reported as possible antioxidant agents that can reduce vascular oxidative stress in cardiovascular events.

**Methods:**

We investigate the influences of haptoglobin, manganese superoxide dismutase (MnSOD Val9Ala), catalase (CAT -21A/T), glutathione peroxidase 1 (GPx-1 Pro198Leu), ACE (I/D) and gluthatione S-transferases GSTM1 and GSTT1 gene polymorphisms against DNA damage and oxidative stress. These were induced by exposing leukocytes from peripheral blood of healthy humans (N = 135) to hydrogen peroxide (H_2_O_2_), and the effects were tested by comet assay. Blood samples were submitted to genotyping and comet assay (before and after treatment with H_2_O_2 _at 250 μM and 1 mM).

**Results:**

After treatment with H_2_O_2 _at 250 μM, the GPx-1 polymorphism significantly influenced results of comet assay and a possible association of the Pro/Leu genotype with higher DNA damage was found. The highest or lowest DNA damage also depended on interaction between GPX-1/ACE and Hp/GSTM1T1 polymorphisms when hydrogen peroxide treatment increased oxidative stress.

**Conclusions:**

The GPx-1 polymorphism and the interactions between GPX-1/ACE and Hp/GSTM1T1 can be determining factors for DNA oxidation provoked by hydrogen peroxide, and thus for higher susceptibility to or protection against oxidative stress suffered by healthy individuals.

## Background

Normal cellular metabolism is well established as the source of endogenous reactive oxygen species (ROS), mainly as a result of normal oxidative metabolism in the mitochondria [1,2]. Because the byproducts of oxidative phosphorylation reactions can diffuse from mitochondria, reach the nuclear DNA and induce damage [3], this cellular process accounts for the background levels of oxidative damage to DNA detected in normal tissue [4,5].

Under normal circumstances, ROS are neutralized by an elaborate antioxidant defense system consisting of enzymes such as catalase (CAT), superoxide dismutase (SOD), glutathione peroxidase (GPx) and numerous non-enzymatic antioxidants [1], [6], [7]. Although a certain level of damage cannot be avoided, oxidative stress can occur when the balance is upset, either by an excessive production of ROS, by deficient antioxidant defenses, or by a combination of both [5]. In such circumstances, ROS may interact with cellular biomolecules, such as DNA, leading to modification and potentially serious consequences for the cell [8]. In this context, the comet assay could be used to evaluate this damage, because it is widely accepted as a standard method for assessing DNA damage type in individual cells [5,9-11]. Although this is not the only way to measure oxidative DNA damage, it is one of the most sensitive and accurate [5]. It is a valuable tool for population monitoring, for example in assessing the role of oxidative stress in human disease, mechanisms of mutagenesis, genotoxicology and ecological monitoring. It is also used to investigate DNA damage and repair in different cell types in response to a range of DNA-damaging agents, as well as monitoring the effects of dietary antioxidants [5,9-11].

Several potentially significant genetic variants related to oxidative stress have already been identified [12,13]. Examples include the glutathione transferase (GST) null alleles and many single nucleotide polymorphisms (SNPs) such as the Val9Ala in the mitochondrial targeting sequence of the MnSOD gene (NCBI, refSNP ID: rs1799725), -21A/T in the promoter region of the CAT gene (NCBI, refSNP ID: rs7943316) and Pro198Leu of the GPx-1 gene (NCBI, refSNP ID: rs1050450). Most of these genetic variants have been reported to result in changes in enzyme levels or activities, which can lead to reduction in protection against oxidative stress [12-19]. In addition, it has been demonstrated that the ability of serum glycoprotein haptoglobin (Hp) to bind free hemoglobin (Hb) in the plasma and block Hb-induced oxidative damage is phenotype dependent [20]. Hp polymorphism has been associated with the prevalence and clinical evolution of many inflammatory diseases, including infections, atherosclerosis and autoimmune disorders [21]; such associations can be explained by functional differences between the phenotypes [20,21]. Moreover, angiotensin I-converting enzyme (ACE) inhibitors have been reported to have beneficial effects on the prognosis and progression of atherosclerosis, suggesting that they can be antioxidant agents that can reduce vascular oxidative stress in cardiovascular events [22]. They also indicate that ACE polymorphisms could be better investigated in the context of oxidative stress, at least those related to cardiovascular risk.

Thus, the aim of this study was to verify, through comet assay, the influences of gene polymorphisms on the oxidative damage to DNA. For this, we investigated the genetic polymorphisms of Haptoglobin, MnSOD (Val9Ala), CAT (-21A/T), GPx-1 (Pro198Leu), ACE, GSTM1 and GSTT1 against DNA damage and oxidative stress induced by exposure to hydrogen peroxide (H_2_O_2_) of leukocytes from peripheral blood of healthy human individuals.

## Methods

### Study design and participants

The sample consisted of 135 individuals of both sexes (55 men and 80 women) and different age groups (17 to 56) randomly recruited in high schools, colleges and universities of Brasília (Federal District, Brazil). It was composed of non-hypertensive, non-diabetic, non-dislipidemic and apparently healthy individuals (inclusion/exclusion criterion), of whom 121 were non-smokers and 14 were smokers. Based on the subjects' self-declared skin color, 29.6% were racially mixed, 38.6% were white, 3.7% were black, 2.2% were Asiatic subjects and 25.9% did not declare their color. The volunteers were informed about the purpose of the study and all of them received a random number.

This study was conducted according to the guidelines laid down in the Declaration of Helsinki and all procedures involving human subjects were approved by the Ethics Committee for Health Sciences Faculty Research of the University of Brasília, number 092/2008. Written informed consent was obtained from all the registered volunteers and all subjects were free to withdraw at any time during the study.

### Procedures and measurements

About 5 mL of peripheral blood was collected by venipuncture using Vacutainer tubes with EDTA as anticoagulant for the comet assay and gene polymorphism analyses. Blood collection was done *in situ*, and blood was then immediately processed for the comet assay.

### Single-cell-gel electrophoresis

The comet assay (alkali method) was performed according to the method developed by Singh et al., 1988 [23], with a few modifications. Briefly, 20 μL of total blood of each sample were mixed with 120 μL of 0.5% low-melting-point agarose in PBS (LMA) (Gibco BRL, Grand Island, N.Y. 14072 USA) at 37°C and pipetted onto eight microscope slides pre-coated with a layer of 1.5% normal-melting-point agarose prepared in phosphate-buffered saline (PBS). Treatment was done with 100 μL hydrogen peroxide (H_2_O_2_) at concentrations of 250 μM and 1.0 mM, for five minutes at 4°C. The control slides were prepared under the same conditions, but without the hydrogen peroxide. Slides were then immersed in a freshly prepared cold (4°C) lysis solution (2.5 M NaCl, 100 mM Na_2_EDTA, 10 mM Tris, pH 10.0-10.5, 1% lauroyl sarcosine, with 1% Triton X-100 and 10% dimethyl sulfoxide added fresh) for 1 hour at 4°C. After lysis, slides were placed in a horizontal gel electrophoresis tank with fresh alkaline electrophoresis buffer (300 mM NaOH, 1 mM Na_2_EDTA, pH >13.0) and left in the solution for 40 minutes at 4°C. Electrophoresis was conducted at 4°C for 30 minutes at 25 V (0.8 V/cm) and 300 mA. Once completed, slides were washed three times with neutralizing solution (0.4 M Tris, pH 7.5), stained with EtBr (20 μg/ml) and analyzed with a Zeiss Axioskop 2 fluorescence microscope (filter 510-560 nm, barrier filter 590 nm) with a total magnification of 400×. One hundred comets on each slide were scored visually by a trained professional as belonging to one of the five classes proposed by Collins et al., 1995 [4], and the DNA damage was calculated according to Jaloszynski et al., 1997 [24]. All experiments were repeated twice using blood samples from different donors. Moreover, in all cases preliminary experiments were performed to single out the best experimental conditions.

### Genotyping of Haptoglobin (Hp), antioxidant enzymes (MnSOD, CAT, GPX1, ACE, GSTM1 and GSTT1

DNA was isolated from the buffy-coat layer by using a Blood genomicPrep Mini Spin Kit (GE Healthcare, Buckinghamshire, England) purification kit, and the samples were stored below -20°C until analysis. DNA samples underwent amplification in an MJ PTC-100 (MJ. Research Inc., Waltham, MA 02451 USA). Hp genotypes were determined by allele-specific polymerase chain reaction (PCR) as described by Yano et al., 1998 [25], while Mn-SOD, CAT and GPx-1 genotypes were determined by polymerase chain reaction (PCR)-based restriction fragment length polymorphism (RFLP) assays performed as described respectively by Mitrunen et al., 2001 [26], Ukkola et al., 2001 [27] and Zhao et al., 2005 [28]. DNA fragments containing I/D polymorphism in intron 16 of the ACE gene were amplified by PCR as previously described by Rigat et al., 1992 [29], using DMSO (dimethyl sulfoxide) as recommended by Odawara et al., 1997 [30], to avoid mistyping of the DD genotype. The glutathione S-transferase (GST) GSTM1 and GSTT1 fragments were amplified simultaneously as proposed by Chen et al., 1996 [31], using β-globin as positive control. The absence of an amplification product combined with the presence of a positive control band (268 bp DNA fragment of β-globin) indicated the null (variant) type for both polymorphisms. The PCR and PCR-based RFLP products were resolved in non-denaturing polyacrylamide gels stained with silver nitrate.

### Statistical analysis

The minimum sample size of each genotype was estimated through a power analysis where the maximum tolerable errors were set at 0.10 for the rare genotypes (such as Hp1F-1F, Hp1F-1S and GPx-1 Leu/Leu) and at 0.05 for the other genotypes. Statistical analysis was carried out using SPSS (Statistical Package for the Social Sciences) version 15.0. Data were expressed as mean ± SEM (standard error of mean) of the percentage of moderate damages (comets of *class *1 and 2) and percentage of elevated damages (comets of *class *3 and 4) and values of p < 0.05 were considered statistically significant. Besides the influences of gene polymorphisms, the influences of sex (55 men and 80 women), use of tobacco and age groups were also treated statistically. The stratification of subjects according to age was done according to their inclusion in one of the following groups: adolescents (15-19 year-olds), young adults (20-40 year-olds) and middle-aged adults (41-56 year-olds), following the age criteria for reference values of biochemical parameters; for clinical purposes some reference values are different for ages up to 19 years old [32].

The continuous variables were tested for normal distribution with Shapiro-Wilk. Differences between sexes and between non-smokers and smokers were evaluated by the Mann-Whitney U test, while possible differences between the groups analyzed were investigated through Kruskal-Wallis test, given that the data presented heterogeneous variability. For the Kruskal-Wallis significant results, Mann-Whitney U test was performed to verify differences between genotypes (2-to-2 comparisons), and the Odds Ratio (OR) with 95% confidence intervals (CI) was calculated to estimate whether genotypes were associated with higher/lower DNA damage. For this, the Pearson chi-square test was applied and differences were considered significant at p < 0.05. To verify differences between the control and treatment slides, the Friedman test followed by the Wilcoxon test were used, respectively for multiple and 2-to-2 comparisons. Differences were considered significant at p < 0.05. The relationship between age groups and DNA damage was analyzed through Pearson correlation test followed by linear regression, and the interactions between two genetic markers in the results of comet assay were analyzed through Multivariate Analyses of Variance.

Allelic and genotypic frequencies were estimated by gene counting, and the goodness of fit of the genotype distribution for Hardy-Weinberg equilibrium (HWE) was tested by chi-square (χ^2^) test. Values of p > 0.05 indicated HWE. Probability (p) values for co-dominant markers (Hp, ACE, MnSOD, CAT and GPx-1) were generated using Genepopweb Statistical Programme version 4.0 http://genepop.curtin.edu.au. For dominant markers (GSTM1 and GSTT1), χ^2 ^test was calculated using a chi-square calculator program. As the PCR method is not suitable for distinguishing between homozygous (+/+, wild type) and heterozygous (+/-), these two groups were considered together (non-null genotypes) and compared with the variant group (null genotypes).

## Results

**Figure 1:**
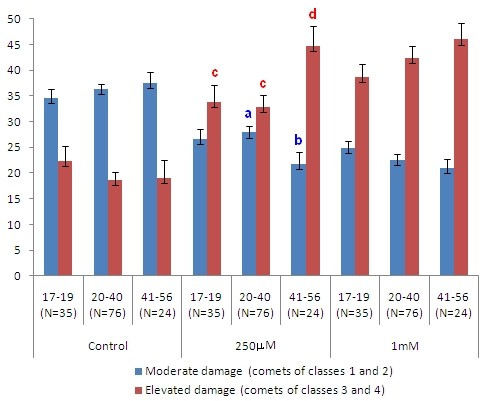
**Influences of age groups (year-olds) on the frequencies (%) of moderate and high DNA damage of the total group**. N = sample size. The data correspond to the means and to the standard error of mean (SEM) of the frequencies (%) of comets of *class *1 and 2 (moderate damage) and of comets of *class *3 and 4 (elevated damage) with respect to the total damages. P-values were generated by the Kruskall-Wallis test. The lower-case letters indicate significant differences between the age groups detected by the Mann-Whitney U test in the 2-to-2 comparisons: for moderate damage, **a **= significant compared to **b**: p = 0.014 in the comparison of 20-40 and 41-56 year-olds; for elevated damages, **c **= significant compared to **d**: p = 0.044 in the comparison of 17-19 and 41-56 year-olds and p = 0.008 in the comparison of 20-40 and 41-56 year-olds.

**Figure 2:**
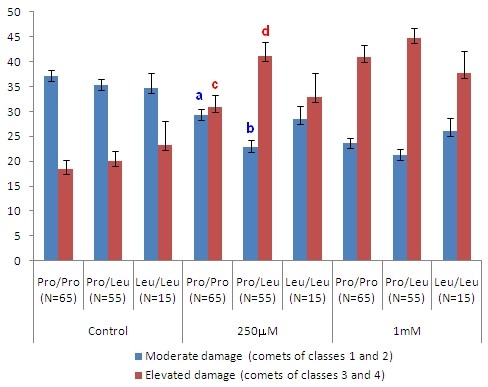
**Influences of GPx-1 gene polymorphism on the frequencies (%) of moderate and high DNA damage of the total group**. N = sample size. The data correspond to the means and to the standard error of mean (SEM) of the frequencies (%) of comets of *class *1 and 2 (moderate damage) and of comets of *class *3 and 4 (elevated damage) with respect to the total damages. P-values were generated by the Kruskall-Wallis test. The lower-case letters indicate significant differences detected by the Mann-Whitney U test in the 2-to-2 comparisons between genotypes: for moderate damage, **a **= significant compared to **b **(p = 0.002); for elevated damages, **c **= significant compared to **d **(p = 0.004).

The final sample size of 135 volunteers obeyed the power calculation for the minimum sample size for Hp1F-1F (N = 6), Hp1F-1S (N = 4) and Gpx-1 Leu/Leu (N = 5). No significant differences were observed between sexes before (control) and after treatment with hydrogen peroxide (H_2_O_2_). Significant differences appeared for the comparisons between age groups of 17-19 and 41-56 year-olds (p = 0.021), 20-40 and 41-56 year-olds (p = 0.036), and between the GPx-1 genotypes Pro/Pro and Pro/Leu (p = 0.012) after treatment with H_2_O_2 _at 250 μM. Concerning age groups, these significant differences were related to the frequencies of moderate damages between 20-40 and 41-56 year-olds (p = 0.014), and to elevated damages between 17-19 and 41-56 year-olds (p = 0.044) and between 20-40 and 41-56 year-olds (p = 0.008) (Figure 1). With respect to the GPx-1 Pro/Pro and Pro/Leu genotypes, the significant differences were related to the both frequencies, moderate (p = 0.002) and elevated (p = 0.004) damages (Figure 2).

There was also a positive significant relationship between age groups and DNA damage after treatment with H_2_O_2 _at 250 μM (p = 0.026). The Odds Ratio (OR) with 95% confidence intervals (CI) was of 0.533 (CI: 0.269 to 1.056) for GPx-1 Pro/Pro (p = 0.070); 2.625 (CI: 1.293 to 5.331) for GPx-1 Pro/Leu (p = 0.007); and 0.468 (CI: 0.151 to 1.450) for GPx-1 Leu/Leu (p = 0.181). No significant differences were observed between the other genetic markers' genotypes or between non-smokers and smokers.

There was a positive correlation between H_2_O_2 _concentrations and DNA-damage levels. The two H_2_O_2 _treated groups were significantly more damaged than the controls in almost all analyses (Friedman, p < 0.05), except for Hp1F-1F (Friedman, p = 0.311). The leukocytes treated with 250 μM of H_2_O_2 _were also significantly less damaged than those treated with 1.0 mM for the total group (Wilcoxon test, p < 0.001), sexes (Wilcoxon test, p = 0.005 for males and p = 0.001 for females) and age group of 20-40 year-olds (Wilcoxon test, p < 0.001). For the genetic markers, the significant differences between treatments with H_2_O_2 _at 250 μM and 1 mM were for Hp 1S-2 (p < 0.001), Hp 2-2 (p = 0.044), MnSOD Val/Val (p = 0.012), MnSOD Val/Ala (p < 0.001), CAT AA (p = 0.005), CAT AT (p = 0.022), CAT TT (0.012), GPx-1 Pro/Pro (p < 0.001), GPx-1 Pro/Leu (0.041), ACE DD (p = 0.011), ACE ID (p = 0.002), GSTM1- (p = 0.002), GSTM1+ (p = 0.001), GSTT1+ (p < 0.001) and for GSTM1-T1+ (p = 0.001) and M1+T1+ (p = 0.006).

When the interaction between two genetic markers was performed, significant results appeared for GPx-1/ACE after treatment with 250 μM of H_2_O_2 _(p = 0.027), and for Hp/GSTM1T1 for leukocytes treated with 1 mM of H_2_O_2 _(p = 0.041). For the interaction GPx-1/ACE, the greatest damage was presented by individuals carrying GPx-1 Pro/Leu and ACE DD genotypes; while the least damage was observed for individuals GPx-1 Pro/Pro and ACE DD. For the interaction Hp/GSTM1T1, major damages were observed for individuals Hp 1F-1F/GSTM1+T1+ and Hp 1F-1S/M1+T1-, while the smallest damages were seen in subjects carrying Hp 1F-1S/GSTM1-T1- and Hp 1F-2/GSTM1+T1+.

Results indicated a significant deviation from Hardy-Weinberg equilibrium (HWE) for the CAT (p = 0.012), MnSOD (p < 0.001), GSTM1 (p < 0.001) and GSTT1 (p < 0.001) loci (Table 1). The CAT locus was appropriate for a heterozygote deficit (p = 0.0085), while the MnSOD locus was for a heterozygote excess (p < 0.001). For GSTM1 and GSTT1, results were compatible with heterozygote deficit, due to homozygous (+/+, wild type) and heterozygous (+/-) being considered together within non-null genotypes, given that the PCR method is not suitable for distinguishing these genotypes. The genotypic distributions of Hp, GPx-1 and ACE loci were in accordance with HWE. MnSOD locus presented a heterozygosity-observed (Ho) value higher than the heterozygosity-expected (He) value, and an F_IS _value (-0.4093) compatible with selection in favor of heterozygotes, while for the CAT locus data of Ho, He and F_IS _value (+0.2141) were compatible with homozygote excess (AA and TT).

**Table 1:** Distribution of Haptoglobin (Hp), MnSOD, CAT, GPx-1, ACE, GSTM1 and GSTT1 allele frequencies, genetic diversity parameters, genotype frequencies and Hardy-Weinberg equilibrium data for chi-square (χ^2^) test.

Genetic Markers	Allele frequencies		Heterozygosity-observed (H_o_)		Heterozygosity-expected (H_e_)		F_IS_ (Inbreeding coefficient)	Genotypes	Genotype frequencies	Number of observed individuals	Number of expected individuals	HWE test (p-values)
**Hp**	Hp*^1*F*^	0.144	0.570		0.594		0.046	**1F-1F**	0.04	6	2.75	0.132
							
								**1F-1S**	0.06	8	13.05	
						
	Hp*^1*S*^	0.333						**1S-1S**	0.12	16	14.89	
							
								**1F-2**	0.14	19	20.44	
						
	Hp*^2^	0.522						**1S-2**	0.37	50	47.17	
							
								**2-2**	0.27	36	36.69	

MnSOD*			0.704		0.498		-0.409	**Val/Val**	0.18	24	37.74	< 0.001
							
	**Val**	0.530						**Val/Ala**	0.70	95	67.51	
						
	**Ala**	0.470						**Ala/Ala**	0.12	16	29.74	

CAT*			0.385		0.488		0.214	**AA**	0.23	31	23.94	0.012
							
	**A**	0.422						**AT**	0.39	52	66.11	
						
	**T**	0.578						**TT**	0.39	52	44.94	

**GPx-1**			0.407		0.432		0.059	**Pro/Pro**	0.48	65	63.27	0.551
							
	**Pro**	0.685						**Pro/Leu**	0.41	55	58.46	
						
	**Leu**	0.315						**Leu/Leu**	0.11	15	13.27	

**ACE**			0.467		0.482		0.034	**DD**	0.36	49	47.88	0.721
							
	**D**	0.596						**ID**	0.47	63	65.24	
						
	**I**	0.404						**II**	0.17	23	21.88	

GSTM1*	**-**	0.622						**Null**	0.62	84	52.23	< 0.001
			-		-		-					
	**+**	0.378						**Non-null**	0.38	51	82.77	

GSTT1*	**-**	0.244						**Null**	0.24	33	8.04	< 0.001
			-		-		-					
	**+**	0.756						**Non-null**	0.76	102	126.96	

*p < 0.05 indicates deviation from Hardy-Weinberg equilibrium, appropriate to a heterozygote deficit for CAT *locus *(p = 0.0085) and a heterozygote excess for MnSOD *locus *(p = 0.0000). For co-dominant markers (Hp, MnsOD, CAT, GPX-1 and ACE) p-values were generated using statistical programme Genepopweb version 4.0 http://genepop.curtin.edu.au; for dominant markers (GSTM1 and GSTT1), they were calculated by a chi-square calculator programme. For GSTM1 and GSTT1 genotypes were considered: Null = -/-; Non-null = +/+ and +/-.

## Discussion

The relatively high concentrations of hydrogen peroxide used in the present study were to provide oxidative stress, since this was a secondary aim of this study. The choices of 250 μM and 1 mM were based on a previous result from studies by our group: the treatment with H_2_O_2 _at 250 μm showed DNA damage similar to that suffered by trained athletes after running a race [33]. Hydrogen peroxide (H_2_O_2_) is an important reactive oxygen species (ROS) that arises either during the aerobic respiration process or as a by-product of water radiolysis after exposure to ionizing radiation [34]. The reaction of H_2_O_2 _with transition metals imposes an oxidative stress condition on cells. This in turn can result in damage to cell components such as proteins, lipids and principally to DNA, leading to mutagenesis and cell death [1,34]. It has been shown that H_2_O_2 _provokes permanent growth arrest and apoptosis in a number of cell types [35]. In this context, our results indicate that oxidative stress, such as that simulated by treatment with H_2_O_2 _at 250 μM, can be more harmful for older subjects. This is in accordance with the literature, since aging is an inevitable biological process and characterized by a general decline in physiological function [36,37]. Moreover, the excessive production of ROS and the consequent imbalance between their concentrations and the antioxidant defense may be related to the aging process [37].

Likewise, since extracellular hemoglobin becomes highly toxic due to the oxidative capacity of its iron-containing heme, which participates in the Fenton reaction to produce reactive oxygen species, and the protective effect of Hp against this oxidative mechanism is phenotype dependent [20,21], our results also indicate that Hp1F-1F can be more protective against oxidative damages to DNA induced by hydrogen peroxide. This is because it was the only genotype where the two H_2_O_2 _treated groups were not significantly more damaged than the controls. Two previous studies by our group corroborate this suggestion [33,38]. The first study, run with a young population group of Brasilia, indicated that among Hp*^1 ^alleles, Hp*^1F ^was the most protective, besides showing that homozygous Hp 1F-1F incurred less DNA damage than other genotypes [38]. In the second study, carried out with runners, one of the main factors that contributed to deviation of the Hp *locus *from HWE was the higher frequencies of Hp1F-1F than the expected [33], indicating that this genotype can be more protective in situations that impose oxidative stress. Since it is well known that polymorphisms in genes involved in xenobiotic metabolism represent a substantial component of individual susceptibility to environmental mutagens and carcinogens [39], the interaction between Hp/GSTM1T1 could also be taken into account. In this context, our results indicated that, under higher hydrogen peroxide concentrations, the protective effect of Hp*^1 ^alleles can depend on the interaction with GSTM1T1 genotypes.

Additionally, synthesis of the antioxidant enzymes is part of the adaptive response triggered by the stress posed by ROS [34]. Glutathione peroxidase 1 (GPx-1, EC 1.11.1.9) catalyses the reduction of hydrogen peroxide to water in the erythrocytes, using glutathione as a reducing substrate [28,40]. Polymorphisms in the GPx-1 gene (locus 3p21.3, OMIM, +138320) have been related to enhanced risk of cancer [41] and some studies have indicated that the Leu variant of GPx-1 gene affects the activity of enzyme GPx-1, which becomes less responsive to stimulation [28,42,43]. In this context, our results indicate that increased oxidative stress can be harmful for Leu allele carriers, since Pro/Leu genotypes presented a significantly increased risk of DNA damage when their leukocytes were exposed to hydrogen peroxide at 250 μM. Although Leu/Leu genotype presented intermediate DNA damage under this hydrogen peroxide concentration, there were no significant differences when it was compared with Pro/Pro or Pro/Leu genotypes, due to higher variability in the responses (higher SEM). Thus, at this hydrogen peroxide concentration, results also indicate that the interaction between GPx-1 and ACE genotypes can be more significant for phenotype manifestation than for GPX-1 polymorphism only.

It has been reported that ACE DD polymorphism is a potent risk factor for myocardial infarction [44]. ACE inhibitors have beneficial effects on the prognosis and progression of atherosclerosis [22,44], suggesting that they may be antioxidant agents that can reduce vascular oxidative stress in cardiovascular events [22]. In the presented context and focusing on DNA damage, our results suggest that higher or lower damage after exposure to hydrogen peroxide at 250 μM was mainly related to the interaction between ACE and GPx-1 polymorphisms rather than to ACE DD genotype. However, different populations apparently responded differently with regard to the appearance and overall impact of the ACE polymorphism on myocardial infarction. One of the potential answers is related to the composition of polymorphisms in different study populations [44].

In the same way, the frequencies of variants, such as the Ala allele of MnSOD gene, T allele of CAT gene and Leu allele of GPx-1 gene vary between ethnic groups [27,28,45]; in Brazil there are few studies that describe these antioxidant polymorphisms. Interethnic differences in the allele frequencies of GST null genotypes and Hp polymorphism have also been documented worldwide and some gradients and intra-ethnic differences have already been reported [18,19,46]. Thus, studies mapping their distribution can be important to gain a better understanding of their biological significance.

The Brazilian population as a whole is very mixed and heterogeneous, primarily as a result of five centuries of interethnic crosses between Europeans, Africans and Amerindians [47], and this miscegenation can influence the distribution of certain polymorphisms. Given that the Federal District was formed by a wide-ranging mixture of migrants from all regions of Brazil [48] its population tends to reflect the constitution of the Brazilian population better than those of other Brazilian regions. In this context, our results suggest a possible adaptive advantage for MnSOD heterozygosis; further, they are in accordance with other results obtained by our group with other population groups of Brasília [33,49]. This is because deviations from Hardy Weinberg Equilibrium might be explained by natural selection [47], and natural selection can act at the level of genes, if particular genotypes allow for increased fitness in specific environments [50]. Concerning CAT gene, the deviation from Hardy Weinberg Equilibrium in favor of homozygosis was not observed in these studies. However, another study that our group carried out with athletes suggested greater efficiency in oxygen transport for individuals carrying variant T allele, because they presented significantly higher mean cell hemoglobin (MCH) values in comparison with other CAT genotypes [51]. Nevertheless, from our current results with comet assay, any selective advantage that may exist is not directly related to protection against DNA damage, given that for this parameter there are no significant differences between MnSOD and CAT genotypes.

## Conclusions

After treatment with H_2_O_2 _at 250 μM there were significant differences between DNA damage in younger and older age groups, indicating that oxidative stress can be more harmful for older subjects. Under similar conditions, GPx-1 polymorphism significantly influenced results of the comet assay, and a possible association between Pro/Leu genotype with higher DNA damage was found. Under increased oxidative stress due to hydrogen peroxide treatment, the highest or lowest DNA damage also depended on the interaction between GPX-1/ACE and Hp/GSTM1T1 polymorphisms. This indicates that these polymorphisms and interactions can be determining factors for DNA oxidation provoked by hydrogen peroxide, and thus for higher susceptibility to or protection against oxidative stress suffered by healthy individuals.

## List of abbreviations

H_2_O_2_: hydrogen peroxide; ROS: reactive oxygen species; CAT: catalase; MnSOD: manganese superoxide dismutase; GPx-1: glutathione peroxidase 1; Hp: haptoglobin; ACE: angiotensin I-converting enzyme; GST: glutathione transferase; SEM: standard error of mean.

## Competing interests

The authors declare that they have no competing interests.

## Authors' contributions

All authors have made a substantial contribution to this study and to the writing and editing of the manuscript. Additional contributions are as follows: ALMV, CKG and MNKG were responsible for the conception and design of the study; CKG and MNKG coordinated this study; ALMV, PCZA, AKA and GSL were responsible for the genotyping and comet assay; CAG was responsible for the statistical analysis of data and ALMV for the interpretation of data as well as for structuring and writing the manuscript. All authors read and approved the final manuscript.
